# Assessing equity: a way to improve sanitation service delivery in South African informal settlements

**DOI:** 10.2166/washdev.2018.166

**Published:** 2018-04-18

**Authors:** S. M. Pan, N. P. Armitage, M. B. van Ryneveld

**Affiliations:** Department of Civil Engineering, University of Cape Town, Rondebosch 7700, South Africa

**Keywords:** equity, informal settlements, sanitation, South Africa

## Abstract

This paper discusses the need to incorporate equity assessment into the planning and monitoring of sanitation service delivery to South African informal settlements. Equity assessment criteria were drawn from literature and a study of sanitation service delivery to informal settlements in three South African municipalities (Cape Town, Johannesburg and eThekwini) over the period 2012-2015. Three key dimensions of equity - resource allocation, access and stakeholder perceptions - were identified. These had eight associated criteria: (1) funds allocated for basic sanitation, (2) number of staff allocated to informal settlements, (3) disparities in access, (4) proportion of functioning sanitation facilities, (5) menstrual hygiene management (MHM) inclusion, (6) access to information, (7) meets users' notions of dignity, and (8) integration of the perspectives of key stakeholders. Key findings of the study indicate that the current focus on reducing service backlogs largely ignores equity and there is a need to better address this through the incorporation of: equity assessments, improving access to information, and the inclusion of marginalised communities in the planning of sanitation services.

## INTRODUCTION

The aim to achieve universal sanitation access in South Africa provides a unique opportunity to set positive regional precedents in sub-Saharan Africa. As one of the wealthiest countries in sub-Saharan Africa (Briceño-garmendia et al. 2008; World Bank 2016) it has an advantage over many neighbouring countries. The country, however, also faces major challenges as it is one of the world's most unequal in terms of income distribution (van der Berg 2010; UN-HABITAT 2012) and access to services.

This paper describes the outcome of a study into how to incorporate equity assessment into sanitation service delivery to South African informal settlements and provides an example of potential applications to improve sanitation services. The working definition of equity used here refers to the ethical concepts relating to notions of social justice, fairness and human rights based on need as a foundation for the distribution of resources (Scott et al. 2012) and power (Oden 2010).

The South African government is committed to providing a baseline level of 'free basic services' (water, sanitation, refuse removal and electricity) to all indigent households (DME 2003; DWAF 2008; Muller 2008), a substantial proportion of whom live in informal settlements. Sanitation as a way of promoting dignity, which has been connected to concepts of urban citizenship, and modernity (Morales et al. 2014: p. 2816; Robins 2014) has been used to advocate a 'rights-based' argument for government-funded sanitation service improvements in informal settlements using the logic that having to use 'unhygienic, inadequate toilet facilities impairs dignity' (Tissington 2011). Thus, inadequate access to sanitation contradicts the constitutional right to have 'dignity respected and protected' (Section 10 of the Bill of Rights (RSA 1996)). Consequently, the national government adopted a Free Basic Sanitation (FBSan) implementation strategy in 2008 (DWAF) which is funded through a combination of national grants and municipal revenue. The FBSan policy supports municipalities in the provision of subsidised basic sanitation services to qualified beneficiaries where a basic service is defined as:

*'The provision of a basic sanitation facility which is easily accessible to a household, the sustainable operation of the facility, including the safe removal of human waste and wastewater from the premises where this is appropriate and necessary, and the communication of good sanitation, hygiene and related practices'* (DWAF 2003).

Despite this policy, however, South Africa was unable to meet the Millennium Development Goal to halve the proportion of the population without access to improved sanitation (WHO & UNICEF 2015). Furthermore, services are often unevenly distributed between different population groups and regions. This has resulted in high levels of dissatisfaction evidenced by wide-spread 'service delivery protests' (Zille 2013; Robins 2014), many of which took place in informal settlements. The connection between the challenge of meeting service delivery backlogs, uneven distribution of services and dissatisfaction with sanitation services in informal settlements warrants a deeper exploration of equity, as set out in this paper.

## METHODS

The most significant criteria to consider in evaluating the equity of sanitation services were drawn from literature (Haughton 1999; Kranich 2005; Oden 2010; Freeman *et al* 2011; Patkar & Gosling 2011; Scott *et al*. 2012; Morales *et al* 2014) and data from fieldwork and interviews collected between July 2012 and May 2015 in Cape Town (CCT), Johannesburg (CJ) and eThekwini (EM) municipalities. These three municipalities are the most populous in the country and have a significant proportion of informal house-holds, ~1 in 5 (Stats SA 2012). Both quantitative and qualitative data were collected. Ethical clearance was obtained from the University of Cape Town's Engineering and Built Environment ethics committee to conduct research using human subjects by demonstrating minimal risk to participants and gaining informed consent. Unstructured interviews were conducted to get a better understanding of the knowledge, opinions and perspectives of stakeholders in different sectors involved with decision-making and steering the development of sanitation services. A 'snowball sampling' method (Morgan 2008) was employed to expand the network of interviewees from initial contacts. In total, 46 people participated: 2 from provincial government, 29 from municipal (local) government, 1 from national government, 1 from academia, 10 from non-governmental organisations (NGOs) and 3 from the private sector. In addition to the unstructured interviews, informal conversations with residents of informal settlements, including two group discussions facilitated by local NGOs, and field observations in 17 informal settlements in Cape Town (7), eThekwini (4) and Johannesburg (6) between 2012 and 2015, were also used to inform the equity assessment. The interviews were recorded, transcribed and then coded according to three 'dimensions' (resource allocation, access and 'perceptions') covering eight criteria that were identified from the interviews assisted by the literature (Table 1).

**Table 1:** Equity assessment criteria

Dimension/Criteria	Indicator
Resource allocation (Household - HH)	
1. Funds allocated for basic sanitation services	ZAR/HH
2. Number of staff allocated to informal settlements	Staff/HH
Access	
1. Measurable disparities in access	Access ratios between genders, urban/rural area; income brackets
2. Proportion of functioning sanitation facilities	Toilets/HH (annual mean)
3. Needs of vulnerable groups considered including MHM	Qualitative
4. Fair decision-making including accessibility to information	Qualitative
Perceptions	
1. Meets users' notions of dignity	Qualitative
2. Perspectives of key stakeholders are integrated	Qualitative

Quantitative data were collected using available records including the national census data and national treasury reports and unpublished reports. Some of the data sought after, such as the location of water and sanitation facilities in informal settlements, could not be obtained for EM due to concerns over information being misused for political agendas, and, in the case of CJ, because the information was not available.

## RESULTS AND DISCUSSION

### Resource allocation

Even though the study was focused on the largest - and arguably the best resourced - municipalities in the country, there was limited disaggregated data publicly available to adequately assess all of the equity indicators (Table 1) identified. Two proposed measures to indicate the equity of resource allocation presented are to (1) consider the number of staff allocated (Scott *et al*. 2008) to water and sanitation services and (2) the budget allocated to FBSan. Estimates for water and sanitation staff per 10,000 people served and the budget allocated per household for FBSan were made from available budget and departmental reports (Table 2).

**Table 2:** Comparison of staffing ratios and budget for Free Basic Sanitation to informal areas

Resource allocation criteria	eThekwini	Johannesburg	Cape Town
Water/Sanitation dept. staff size (staff/capita) (Total population)	9:10,000 (3,442,361)	6:10,000 (4,434,827)	11:10,000 (for entire city);4:10,000^a^(for informal settlements); (3,740,025)
2013/14 Estimated budget allocated to FBSan (ZAR/HH); (Total # of HHs); (% Informal HHs)	1,486; (956,713); (21%)	253; (1,434,856); (19%)	386; (1,068,572); (22%)

*Sources:* HDA (2013a, 2013b), Crous (2014), EWS (2014), CJ (2014), CCT (2014, 2015) and EM (2015); data from Johannesburg Water covering water and sanitation in informal settlements from 2013 to 2014.

a Oniy Cape Town had a separate unit dedicated to informal settlements; therefore, it was not possible to determine staff allocation focused on informal settlements for EM and CJ.

In terms of available staff resources, CCT had the highest number of staff per capita of the three municipalities. EM, however, had the highest number of registered professional engineers in water services of all the metropolitan (Category A) municipalities (municipalities with over 500,000 voters and who coordinate their own service delivery) and was rated the highest of all metropolitan municipalities in terms of senior technical staff with the appropriate skills (SALGA & WRC 2014). EM also allocated the most funds to support FBSan services in terms of ZAR/household, including the large-scale roll out of communal ablution blocks (Crous 2014), but the allocation also included funds for some low-income formal households that receive FBSan. (*Note:* Only EM clearly indicated different budget lines for FBSan for formal and informal households, so the values for CJ and CCT are likely underestimates.) It is important for municipalities to remain vigilant about equitable distribution of resources to address service inequalities.

### Access

**Figure 1:**
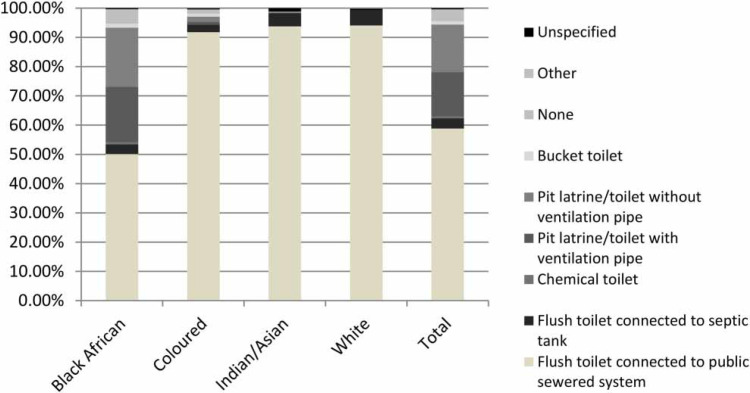
Sanitation facility access by population group represented by the head of household (Stats SA 2014).

**Figure 2:**
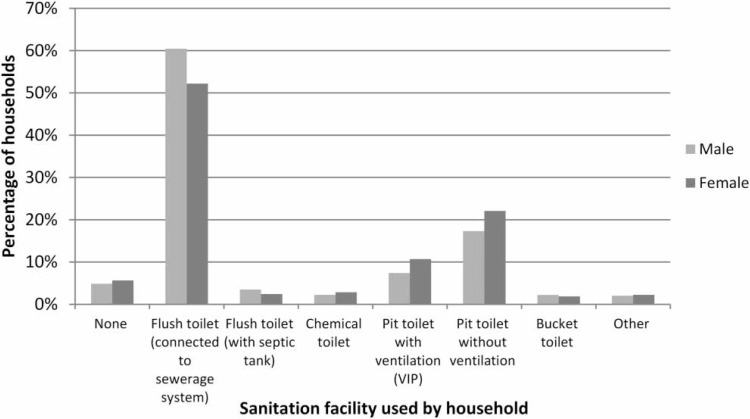
Sanitation facility access by gender of household head (after Stats SA 2013).

**Figure 3:**
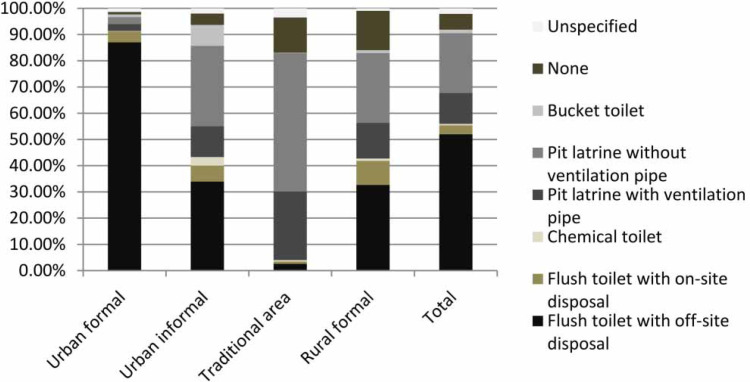
Sanitation facility access by settlement type (after Stats SA 2011).

In terms of access to basic sanitation services across different demographic groups, the assessment was conducted at a national scale; while the rincipal author intended to compare access across the selected municipalities, municipalities used different categories of sanitation facilities which made this difficult. Although national census statistics on sanitation did not include the condition of the sanitation facility, they did indicate some of the disparities that need to be considered, which often correlated to race, gender and where a person lived. Johannesburg had the highest percentage of households overall with access to a 'functioning basic sanitation facility' defined as a 'Flush toilet connected to a public sewerage system or septic tank or a pit latrine with ventilation pipe' (Stats SA 2015) according to the General Household Survey conducted by Stats SA, but Cape Town had the highest percentage of informal households with access to basic sanitation. Menstrual hygiene management (MHM) inclusion, assessed during field visits, was taken as a proxy indicator for including the needs of vulnerable groups. Ease of access to information on sanitation facility coverage statistics and planned development was used as a proxy indicator for determining fair decision-making, which was assessed based on data from the focus group discussions and interviews. There is a measurable disparity between households in different demographic groups (Table 3: Indicators 1 and 2), which will be described further in Figures 1-3.

**Table 3:** Comparison of access to sanitation

Access criteria	eThekwini	Johannesburg	Cape Town
1. Measurable disparities in access (across race, gender, settlement type)	Female-headed black African households in traditional areas and urban informal settlements least likely to have access		
2. Percentage of informal HHs with a ‘basic sanitation facility^a^’; (percentage of all HHs with a ‘basic sanitation facility’)	37.4 (83.5)	41.9 (96.9)	53.2 (91.8)
3. Needs of vulnerable groups considered including MHM	No	No	No
4. Fair decision-making including accessibility to data	No	No	No

*Sources:* Truyens et al. (2013), CCT (2014), EM (2014) and Stats SA (2014, 2015); data from Johannesburg Water covering water and sanitation in informal settlements from 2013 to 2014.

a Chemical, container and bucket toilets were not considered to meet standards for ‘basic sanitation’ by the author since they did not hygienically separate users from excreta.

Figure 1 shows the type of sanitation facility used by different population groups (Stats SA 2014), and demon-strates the disparities that exist between different racial groups, e.g., the group with the highest proportion of the population in South Africa with below basic standards for sanitation facilities identifies as 'Black African'. Addition-ally, there is some level of association between the gender of the head of the household and access to a sanitation facility, e.g., the odds of a female-headed household lacking access to any form of sanitation are 1.2 times higher than for male-headed households (Figure 2). There are also noticeable disparities in the type of sanitation facility between urban and rural areas and formal and informal areas, as shown in Figure 3. People living in urban formal areas are the most likely to have access to at least a basic level of sanitation service or higher, while people living in traditional areas are the least likely. Traditional areas may include peri-urban areas in municipalities such as EM, which incorporated areas formerly considered as 'home-lands' under the apartheid government, which were areas designated for black South Africans and part of the government's strategy to remove black South Africans from urban areas. They were designated as separate administrative regions to the rest of the country or 'white South Africa' and reinforced segregationist policies. Although urban informal areas appear to have the second highest percentage of people with access to a basic level of sanitation service, access is more likely to be in the form of shared sanitation facilities than in other settlement types.

While the majority of households in South Africa have access to a basic level of sanitation, there is a noticeable dis-parity in access related to race, sex and where a person lives, with black African, female-headed households and those located in traditional and urban informal areas the most likely to lack access to basic sanitation. While conclusions about the underlying causes of disparities cannot be drawn from the data, observable disparities within certain demographic groups and geographic regions indicate that they need more assistance than others to improve access to sanitation services.

Another issue that needs to be addressed is MHM (Criteria 3 under Access in Table 1). Part of the issue is that MHM overlaps with solid waste management, which is typically managed separately from water and sanitation services. There is also a need to 'sensitise engineers, planners and water managers regarding infrastructural design that supports MHM' (WIN-SA 2012). Privacy concerns as well as cultural preferences may lead women to deal with MHM at home rather than at communal facilities (Sommer *et al*. 2015). EM has collaborated with various partners to research MHM in the municipality (Truyens *et al*. 2013), which indicates some level of awareness among municipal sanitation project planners. However, options to support MHM at communal facilities were not mentioned by any of the interviewees, and only one of the facilities (Diepsloot pilot project in CJ) out of 17 sites visited across the three municipalities had receptacles for disposal of menstrual waste located within the toilet cubicle or facility for female users. (Note: Outside of the Cape Town informal settlements, site visits were only made once, therefore a longitudinal study was not possible in most cases.)

One of the institutional sanitation service delivery challenges is coordination between various institutions within the municipality as well as with other stakeholders in relation to data synchronisation and sharing, e.g., informal settlement boundaries may differ between different departments' databases. Advocacy NGOs such as SJC (CCT based) and Abahlali base Mjondolo (EM based) have complained about municipalities' reluctance to share data related to services in informal settlements, which was also experienced by the primary author during the research process. Two of the three municipalities declined to share GIS data for the location of informal settlement water and sanitation services, although backlog figures were shared. Part of the issue is that municipalities want to ensure that data are accurate and have been audited prior to sharing, but there is also a reluctance to share data for fear of political back-lash and biased analysis for personal or political agendas, which makes collaboration between different stakeholder groups difficult. The failure to publish reports related to sanitation infrastructure, or lengthy delays in publishing, is an issue that needs to be addressed if service delivery is to improve (Muller 2016). Insufficient public access to information can also be considered as a general equity concern, which was noted by non-government stakeholders.

### Perceptions

Of the three equity dimensions described in this research - resource allocation, access and perceptions - perceptions of dignity and the integration of multiple stakeholders' perceptions into sanitation service programmes were the most difficult to define and measure (Table 4). What constitutes a 'dignified' form and level of service (LoS), which typically in South Africa is associated with a different sanitation technology provided to households on an individual or shared basis, also referred to as a 'rung on the sanitation ladder' (Potter *et al*. 2011), in a system with varying levels of service based on factors that are not in the control of users may lead to ambiguity and a potential disconnect 'between sanitation expectations' and 'the practices required by proposed sanitation solutions' (Morales *et al*. 2014).

**Table 4:** Comparison of perceptions towards sanitation

Perception criteria	eThekwini	Johannesburg	Cape Town
1. Meets users' notions of dignity	No	No	No
2. Perspectives of key stakeholders are integrated	No	No	No

*Sources:* Masondo (2010), Roma *et al*. (2013) and SAHRC (2014). In addition to these sources, the author's fleldwork, interviews and focus group discussions were used to evalúate perceptions.

Some excerpts from conversations with individuals from different stakeholder groups highlight some of the ambiguity related to what constitutes a 'dignified' and equitable sanitation service and why there is a strong perception of inequitable sanitation services across the municipalities (Table 5).

**Table 5:** Quotations from sanitation stakeholders relating to perceptions of equitable sanitation services (from interviews conducted between 2013 and 2015)

Municipal official	NGO employee	'Social entrepreneur'	Informal settlement residents
'So, from an equity point of view we're trying to make sure that… provision is made to everyone. But the quality, the type, the technology will depend on the settlement conditions as well… **It's not that we're discriminating** against those people, **but it's the settlement conditions and where they are.'**	'If you come in from outside and are not willing to use the toilet or drink the water, the **message that you are sending is contradictory.'**	'[There is] a **sanitation marketing** push [which is] a good answer to financia! aspects of sustainability because people make **informed decisions** based on purchasing power about fu!! costs including running costs, [but] there's **not much to do with equity…'**	'We don't expect everything for free. **We are not like animals.''They** [the government] **will leave us there** [in backyard shacks].'

These excerpts cannot paint the full picture of what equitable and dignified sanitation should be defined as; how-ever, they do indicate some general attitudes and perceptions gleaned from interviews and discussions with different key stakeholder groups. Municipal officials inter-viewed typically expressed a sense of technical pragmatism in relation to servicing informal settlements, whereas NGO employees tended to focus on government shortcomings (or apathy) and social issues that could be addressed through sanitation services. Unsurprisingly, sanitation-related 'social entrepreneurs' focused on ways to expand the sanitation market and acknowledged the need to promote equity, but implied that that was primarily a government responsibility. Finally, perhaps the most telling in terms of perceptions of (in)equity, were conversations with informal settlement residents who emphasised a basic desire not to be ignored and, crucially, to be treated like everyone else. Finding a way to incorporate and address concerns demonstrated by these various perspectives, particularly those of informal settlement residents, in the design of sanitation service delivery programmes will be critical to improving equity and supporting the improvement of sanitation services in informal settlements.

Common perceptions of inequity observed across all three municipalities included the following:

(i) dry sanitation is perceived as inferior to waterborne sanitation by many informal settlement users; water-borne systems would be preferred if given the option;
(ii) decision-makers who may never have used alternatives to waterborne sanitation systems before are insensitive to the lived reality of informal settlement residents;
(iii) providing different sanitation systems in the same or neighbouring settlements can lead to tension betweenresidents and can be perceived as preferential treatment.

These various perceptions should inform future sanitation programme planning to help address areas of concern, to clarify misunderstandings and to build common ground between different stakeholder groups. In South Africa, due to government subsidisation of sanitation services through policies like FBSan, local government tends to be the most authoritative decision-maker compared to other stakeholders (NGOs, CBOs, contractors, residents). Residents may be consulted to choose from a generally pre-determined set of sanitation options or to locate facilities, but they are not typically the final decision-makers.

### Limitations and obstacles encountered in the research

The data that this research draws from should only be considered as a 'snapshot' of an extremely dynamic situation. Additionally, although the author reached out to study participants after concluding the field research and interviews to check for consistency and to report preliminary findings, no responses were received. Among multiple obstacles encountered in carrying out this research, two in particular stood out:

(i) the difficulty of disaggregating financial information between water and sanitation projects or formal andinformal areas;
(ii) government officials restricting access to information for fear of negative political repercussions.

## CONCLUSIONS AND RECOMMENDATIONS

This paper describes how considerations of equity could be used to improve sanitation service delivery in South African informal settlements. Three key dimensions - resource allocation, access and stakeholder perceptions - breaking down into eight criteria of equity were identified through literature, fieldwork and interviews in three of the largest South African municipalities. Sanitation services in South Africa are both unequal and inequitable between different demographic groups as compared across race, gender of head of household and settlement type, and were often perceived negatively by informal settlement residents. Informal settlement residents during site visits were sensitive to differences in the type of sanitation technology implemented between formal and informal areas and within or between settlements (inter- and intra-settlement); however, national and municipal monitoring and evaluation frameworks primarily focus on meeting the water and sanitation service delivery backlogs, i.e., number of households receiving below a basic level of service or with no service. These are generally measured as the absence or presence of a facility. The presence of a sanitation facility, however, does not automatically guarantee access or a quality service if equity criteria are not taken into account. 'Fairness also demands remedies to redress historic injustices that have prevented or diminished access in the first place' (Kranich 2005), which demands an assessment of equity into the planning and implementation of sanitation systems to vulnerable groups. Equity requires that consideration be given to the quality of services, access to information, which helps ensure that people are treated openly and fairly particularly as it pertains to decision-making (Haughton 1999), and explicit consideration of the needs of vulnerable groups within a defined spatial unit, e.g., within a municipality or a province. While it may not be possible or necessary for every municipality to measure all of the criteria identified in this research, key criteria could be selected to highlight areas of inequity within a municipality or between different sub-units, e.g., as part of municipal Key Performance Indicators for service delivery or the Municipal Benchmarking Initiative (SALGA & WRC 2014).

'Some for all, forever' (DWAF 1994; RSA 1997) is a core tenet of water and sanitation service policy in South Africa, but the 'some' (as in the level of service) also needs to match local resources and needs to meet criteria for equity, some of which are proposed in this paper. There should also be a vision to increase the service levels for 'all', with priority for national and local government to bring those lower down up to the level of those higher up rather than perpetuating differentiated levels of service indefinitely. A primary goal of sanitation services across the municipality should be to reduce inequity between residents living in informal and formal areas and different demographic groups in the dimensions of equity described. This research is intended to help generate discussion, to provide a tool for describing inequity and sanitation and to catalyse action rather than to prescriptively recommend indicators to use for measuring inequity. Finally, as the analysis of different stakeholders' perceptions of equity indicated, local governments need to find more ways to engage marginalised communities throughout the process of service delivery, for example, through meaningful and inclusive public participation with-out limiting solutions to predetermined technologies, and to avoid treating informal settlements and their inhabitants as anomalies in a 'formal' city.
